# VSI_microfluidics engineering

**DOI:** 10.1016/j.mex.2021.101479

**Published:** 2021-08-06

**Authors:** Kejun Wu, Huaying Chen

Microfluidics is a growing research field that requires a working knowledge of physics, biology, chemistry, and engineering. It has the potential to influence subject areas from chemical synthesis and biological analysis to optics and information technology. However, the impact of microfluidic systems will only become apparent when everyone has easy access to use it. At the current stage, however, detailed information relating to experimental design, troubleshooting, data analysis, etc. for the microfluidic system is still limited in published literature, which may inhibit the broad application of this technology.

The list of papers attached to this SI collection provides essential guidelines for the fabrication of novel microfluidic systems for various applications, such as:

- Fabrication of multi-layer microfluidic chips, control of the membrane deflection to screen cell size, and printing of single cells
- Microfluidic devices enabled colorimetric whole blood glucose sensing assay without the need for blood cell separation
- Coiled flow inverter microreactor for controlled production of metal-organic frameworks
- Method for the precise measurement of mechanical properties of single cells deformed to a large extent
- Methods for the use of microfluidics to model embryonic circulation using differentiated pluripotent stem cells
- Using commercially available microfluidic systems for the formation of tunable particle-size droplets loaded with growth factors
- Colorimetric technique of resazurin for mass transfer characterization

Overall this SI should be of interest to researchers who want to establish new experimental rigs and protocols using microfluidic systems.

Finally, as Guest Editor of this VSI, we would like to thank all the authors for their excellent contribution, all the reviewers for their time and valuable comments and the Methods X team for their professional supports.

